# Department of Agriculture Advises That Milk Be Pasteurized at Low Temperatures

**Published:** 1913-09

**Authors:** 


### Department of Agriculture Advises That Milk Be Pasteurized At Low Temperatures.

In order to determine the best way of pasteurizing milk so as to kill the disease germs and yet not give the milk a cooked flavor or lessen its nutritive value, the Department of Agriculture, through its Dairy Division, has been conducting a series of experiments, treating milk at different temperatures and for different lengths of time. According to the report on these experiments in Bulletin 166 of the Bureau of Animal Industry, when milk is pasteurized at 145° F. for thirty minutes the chemical changes are so slight that it is unlikely that the protein (muscle-building element) or the phosphates of lime and magnesia are rendered less digestible than they are in raw milk.

Moreover, from a bacteriological standpoint, pasteurizing at low temperatures is found to be more satisfactory than pasteurizing at high temperatures. According to Bulletins 126 and 161, where low temperatures are used the majority of bacteria that survive are lactic acid organisms which play an important part in the normal souring of milk. When milk is efficiently pasteurized at high temperatures, the bacteria which survive are largely of the putrefactive kinds, and milk so treated is kept for any length of time has a tendency to rot instead of sour. From the standpoint of economy, the technologist of the Dairy Division finds that pasteurizing at low temperatures calls for less heat. It is found that it takes about 23½ per cent less heat to raise milk to the temperature of 145° F. than to a temperature of 165° F. A similar gain is a saving of the ice needed, because it will require 23½ per cent more refrigeration to cool milk to the shipping point when it is pasteurized at the higher temperature. The Department, therefore, recommends that "When market milk is pasteurized it should be heated to about 145° F. and held at that temperature for thirty minutes."

